# Supplementary material for: Sodium Citrate Alleviates Virulence in Pseudomonas aeruginosa
Source: Microorganisms. 2022 May 18;10(5):1046. doi: 10.3390/microorganisms10051046 (PMC9145658; doi:10.3390/microorganisms10051046)
Supplement: Supplementary file 1 [file microorganisms-10-01046-s001.zip › microorganisms-1710287-supplementary.pdf]

Table S1. The mean absorbances of pyocyanin in citrate treated and untreated culture supernatants at 691 nm.

Control PAO1	Citrate 4%	Citrate 5%
0.313	0.071	0.064
0.326	0.067	0.054
0.315	0.067	0.059

Table S2. The mean absorbances of biofilm cells in citrate treated and untreated culture supernatants at 590 nm.

Control PAO1	Citrate 4%	Citrate 5%
2.103	0.554	0.487
2090	0.525	0.505
2.133	0.525	0.525

Table S3. Fold expression of QS-encoding genes in the presence or absence of 5% citrate

**Gene**	Untreated POA1			Citrate-treated PAO1		
rhlI	0.41	0.45	0.43	-2.90	-3.22	-2.94
rhlR	0.12	0.25	0.24	-3.50	-3.80	-3.75
lasI	0.32	0.40	0.39	-4.53	4.87	-4.62
lasR	0.50	0.63	0.61	-3.90	-4.52	-4.40
pqsA	0.62	0.70	0.65	-2.61	-2.80	-.2.65
pqsR	0.35	0.45	0.46	-4-01	-4.40	-4.23

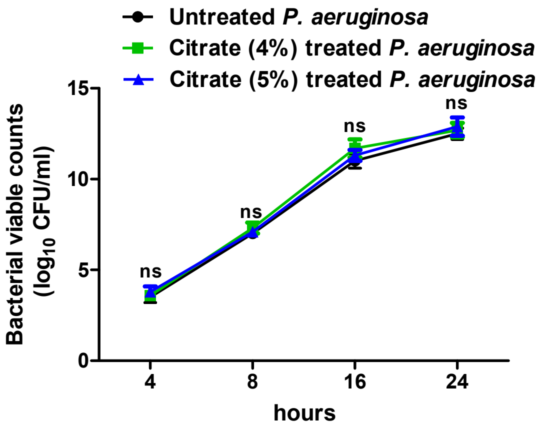

Figure S1. Viable count of the bacterial cells in the presence or absence of citrate after several time intervals. There was no significant effect of sodium citrate on bacterial growth.
